# Comparison of Rapid Diagnostic Test, Microscopy, and Polymerase Chain Reaction for the Detection of *Plasmodium falciparum* Malaria in a Low-Transmission Area, Jazan Region, Southwestern Saudi Arabia

**DOI:** 10.3390/diagnostics12061485

**Published:** 2022-06-17

**Authors:** Aymen M. Madkhali, Ahmad Hassn Ghzwani, Hesham M. Al-Mekhlafi

**Affiliations:** 1 Department of Medical Laboratory Technology, Faculty of Applied Medical Sciences, Jazan University, Jazan 45142, Saudi Arabia; 2 Medical Research Centre, Jazan University, Jazan 45142, Saudi Arabia; ahmad.xixix3@gmail.com; 3 Department of Parasitology, Faculty of Medicine and Health Sciences, Sana’a University, Sana’a 1247, Yemen

**Keywords:** malaria, microscopy, rapid diagnostic test, PCR, infectious diseases, Jazan, Saudi Arabia

## Abstract

This cross-sectional study aimed to assess the performances of a rapid diagnostic test (RDT)—the AllTest Malaria p.f./p.v., microscopy, and nested polymerase chain reaction (PCR) for diagnosing *Plasmodium falciparum* malaria in 400 febrile patients from a low-transmission region (Jazan) in southwestern Saudi Arabia. Diagnostic performance of all three methods was compared using microscopy and nested PCR as reference methods. Overall, 42 (10.5%), 48 (12.0%), and 57 (14.3%) samples were found positive by microscopy, RDT, and PCR, respectively. With PCR as reference method, the RDT showed higher sensitivity (79% vs. 71.9%), similar specificity (99.1% vs. 99.7%), and better NLR (0.20 vs. 0.27) and area under the curve (89.0% vs. 85.8%) than microscopy. The sensitivity of RDT and microscopy decreased as age increased, and false negatives were associated with low parasite density. In addition, the sensitivity of RDT and microscopy was higher in non-Saudi than in Saudi participants. Against microscopy, both RDT and PCR showed high sensitivity (83.3% vs. 97.6%), specificity (96.4% vs. 95.5%), and NPVs (98.0% vs. 99.7%), but reduced PPVs (72.9% vs. 71.9%), respectively. The results showed that the performance of the AllTest Malaria p.f./p.v RDT was better than that of microscopy in diagnosing *P. falciparum* malaria among febrile patients in the Jazan region when nested PCR was used as the reference. However, further studies are required to assess malaria diagnostic methods among asymptomatic individuals in the region.

## 1. Introduction

Malaria is a leading cause of death in developing countries, particularly in tropical and subtropical regions [1]. In 2020, 241 million malaria cases and 627,000 associated deaths were reported worldwide, with approximately 95% of the cases in the African region [2]. This burden challenges the World Health Organization’s (WHO) global technical strategy for malaria 2016–2030, which aims to reduce malaria case incidence and mortality rates globally by at least 90% by 2030 [3]. Malaria in humans is caused by four *Plasmodium* species transmitted by female *Anopheles* mosquitoes, namely *Plasmodium falciparum*, *P. vivax*, *P. malariae,* and *P. ovale*. In addition, *P. knowlesi*—a zoonotic *Plasmodium* species that naturally infects macaques, causing simian malaria—has been identified as a fifth species that causes malaria in humans. Naturally acquired human *P. knowlesi* cases have been reported in many parts of southeast Asia, particularly Malaysia [4,5].

A prompt, accurate diagnosis followed quickly by effective treatment is critical for effective management and surveillance of malaria; these are considered the main pillars of the global technical strategy for malaria 2016–2030 [3,6]. Methods for diagnosing malaria include microscopy, rapid diagnostic tests (RDTs), and amplification of nucleic acids-based assays such as polymerase chain reaction (PCR) and loop-mediated isothermal amplification [7]. Microscopy is the gold standard used for decades to diagnose malaria. It is still the primary method in many healthcare settings globally. Microscopic examination of Giemsa-stained thick or thin peripheral blood smears remains the superior diagnostic method for identifying species and stages of the *Plasmodium* parasite and estimating parasite density. However, the sensitivity and specificity of microscopy depend on the quality of the stained slide and the competency of the microscopists [8]. Moreover, microscopy is not as effective with sub-microscopic malaria (infections with very low parasite density) and mixed-species infections [9,10].

Rapid diagnostic tests are easy to use, fast, cost-effective, and field-deployable tools for malaria diagnosis [11]. Three antigens are usually the targets for commercially available RDTs, namely *P. falciparum* histidine-rich protein 2 (Pf-HRP2), lactate dehydrogenase (LDH), which can be either pan-specific (i.e., genus-specific) or species-specific (P.f.-specific or P.v.-specific), and genus-specific aldolase which detects all *Plasmodium* species [12]. The first RDT was developed in the 1990s and quickly became essential for managing, controlling, and eliminating malaria worldwide [13,14]. However, RDTs have limitations when detecting asymptomatic or low-density infections as well as those from parasite strains that have deletions in the genes encoding HRP2 or HRP3, its structural homologue [15,16,17,18].

In addition, PCR is highly sensitive (2–5 parasites/μL) compared with RDTs (>100 parasites/μL) and microscopy (50–500 parasites/μL) [19,20]. Therefore, PCR is increasingly used for quality control and are useful tools for epidemiological studies mapping sub-microscopic malaria [15,21]. However, PCR is expensive, require qualified personnel, and have a long turn-around time; thus, they are impractical for use in the field or clinical settings, particularly in resource-limited countries [22].

In Saudi Arabia, the national malaria control programme, established in 1948, has achieved remarkable success in reducing the annual number of malaria cases and controlling autochthonous and indigenous malaria transmission. This has been achieved through some control and elimination strategies that include annual indoor residual spraying, long-lasting insecticide-treated nets, and proper management of infection, with individual case follow-up and reactive surveillance [23]. Currently, Saudi Arabia has nearly eliminated malaria, but the disease is still endemic in the southwestern part of the country, specifically in the Jazan and Aseer regions. In the Jazan region, malaria transmission is low, and the incidence rate has been substantially reduced; the lowest number of annual cases (499) was reported in 2014 [24]. However, cases increased after 2014, with 3022 reported in 2020 [24,25]. Malaria is diagnosed using RDTs by malaria control personnel and public and private healthcare facilities. Blood smears are prepared for RDT-positive cases for further examination by microscopy. Eliminating malaria would not be possible without accurate diagnosis; however, data on the performance of malaria diagnostics used in the Jazan region are limited. Therefore, this study aimed to evaluate the performance of RDTs and microscopy—the two main diagnostic methods used in Saudi Arabia—and nested PCR techniques among clinically suspected malaria cases in a low-transmission setting.

## 2. Materials and Methods

### 2.1. Study Design

A cross-sectional study was used to compare three techniques used in malaria parasite detection in the Jazan region. The malaria cases included in this study were detected through passive case detection among clinically suspected cases (febrile patients) who presented at healthcare facilities in selected governorates of the Jazan region.

### 2.2. Study Settings

The Jazan region is in the southwest of the Kingdom of Saudi Arabia, 1100 km from the capital, Riyadh. It is the smallest region in the country, with a total land area of approximately 11,671 km^2^ and a population of approximately 1.8 million [26]. The region comprises 17 governorates in three zones: a highland zone at an elevation of over 2500 m; a hill zone at 400–600 m; and a coastal plain below 400 m [27]. The region contains many valleys, a few small rivers, and approximately 15 dams to provide water for drinking and irrigation. Although malaria transmission in Jazan has been substantially reduced, malaria is still considered endemic, with *Anopheles arabiensis* the main vector [28]. Until 2007, chloroquine alone or with sulfadoxine-pyrimethamine was the first-line treatment for uncomplicated *P. falciparum* malaria. This was replaced by artemisinin-based combination therapy, with artesunate plus sulfadoxine-pyrimethamine and a combination of artemether-lumefantrine used as first- and second-line treatments [29]. In addition, parenteral artesunate and artemether are the first- and second-line treatments for severe *P. falciparum* malaria [29].

Blood samples were collected from febrile patients (suspected malaria cases) presenting at selected hospitals in the study area. All patients were enrolled regardless of their nationality, age, or sex. The participants’ demographic data were collected from their medical records.

### 2.3. Blood Sampling

Approximately 2–3 mL of venous blood was collected from each patient into an EDTA tube clearly labelled with the patient’s reference number, name, sex, and age. Directly after collection, blood samples were examined using a malaria RDT. Thin and thick blood films were prepared on clean, labelled glass slides and stained with diluted Giemsa stain. In addition, blood spots were prepared on clean, labelled 3MM Whatman^®^ filter papers (Whatman Int. Ltd., Cat. no. 3030-917, Maidstone, UK). The prepared blood spots were kept in individual zipped plastic bags at 4–6 °C and used for molecular examination.

### 2.4. Microscopy

The thin and thick blood smears were stained for 45 min with 3% buffer-diluted Giemsa stain solution (pH 7.2) and then examined for *Plasmodium* parasites under a 100 × objective. The thick smears were used to assess the presence of malaria infection and parasite density, while the thin smears were examined to identify the parasite species and stages. At least 200 high power fields of the thick smear had to be examined before a sample was considered negative [30].

For positive smears, parasite density was estimated by counting the asexual stages against 300 WBCs and then multiplying by 25, assuming the mean total WBC count of any human being is 8000 cells per 1 μL of blood [30]. The parasite density was categorised into four groups as follows: <100 parasites/μL; 100–999 parasites/μL; 1000–9999 parasites/μL; and ≥10,000 parasites/μL [31]. About 25% of all slides were randomly selected and re-examined by another expert microscopist for quality control.

### 2.5. AllTest Malaria p.f./p.v. Rapid Test Cassette

This study used AllTest Rapid Test—Malaria p.f./p.v. kits, product code IMPV-402 (Hangzhou AllTest Biotech, Hangzhou, China). This RDT detects *P. falciparum*-specific HRP2 on the P.f. test line and *P. vivax*-specific pLDH on the P.v. test line. A third line is used for positive control. A sample was considered negative if only one band appeared on the control, whereas the presence of another band either in the P.f. or P.v. area together with the control band was considered a *P. falciparum-* or a *P. vivax*-positive result, respectively. Furthermore, when all three bands (a control band and two test area bands) appeared simultaneously, a *P. falciparum*/*P. vivax* mixed infection was recorded. If no control band appeared, the test was considered invalid.

### 2.6. Molecular Analysis

Following the manufacturer’s instructions, DNA was extracted using a Qiagen blood and tissue kit (QIAGEN, DNeasy^®^ Blood & Tissue Kit, Cat. no. 69506, Hilden, Germany). Using a sterile puncher (6 mm diameter), one or two discs of the dried blood spots were cut, placed into a 1.5 mL microcentrifuge tube, and processed for DNA extraction; DNA was eluted using 50 µL AE (10 mm Tris-Cl; 0.5 mm EDTA; pH 9.0) elution buffer and kept at −20 °C until used. The samples were examined using a conventional nested PCR assay using different oligonucleotide primers based on *Plasmodium* 18s rRNA genes following an established protocol [32]. This assay allows the identification of the four major human malaria parasite species (*P. falciparum*, *P. vivax*, *P. malariae,* and *P. ovale*).

### 2.7. Data Analysis

Data were analysed using IBM SPSS version 20 (IBM Corp., New York, NY, USA). The performances of microscopy, AllTest Malaria RDT and PCR in diagnosing malaria were evaluated using the following indicators: sensitivity, specificity, positive predictive value (PPV), negative predictive value (NPV), negative likelihood ratio (NLR), positive likelihood ratio (PLR), and accuracy. These indicators were calculated using the Medcalc^®^ online calculator (https://www.medcalc.org/calc/diagnostic_test.php, accessed on 30 March 2022) and presented with corresponding 95% confidence intervals (CI). In addition, kappa statistics were used to assess agreement between the tests. The area under the curve (AUC)—a powerful way to summarize a diagnostic test’s overall accuracy—was evaluated using the receiver operating characteristic (ROC) curve analysis. A *p*-value of < 0.05 was considered significant.

## 3. Results

**Figure 1:**
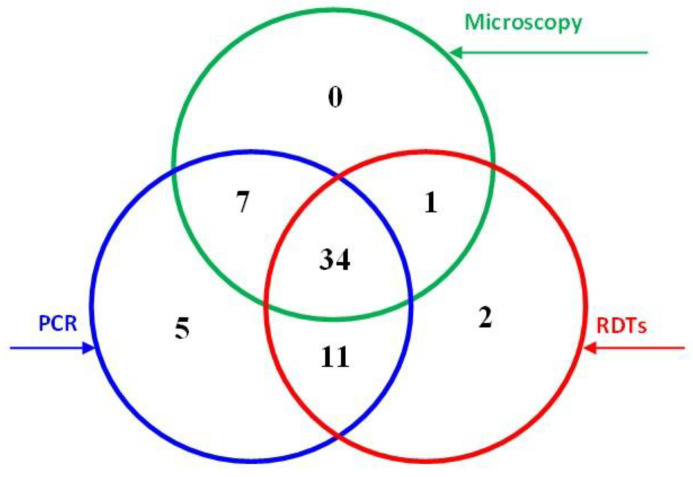
Malaria detection results obtained by light microscopy, RDT, and PCR techniques.

This study tested 400 blood samples using light microscopy of Giemsa-stained blood films, RDT (AllTest Rapid Test—Malaria p.f./p.v. kit) and nested PCR. The results are summarised in Figure 1 and Table 1; 60 samples were found positive for *P. falciparum*, with 34 of these found positive by all three methods. Overall, 42 (10.5%), 48 (12.0%), and 57 (14.3%) samples were found positive by microscopy, RDT, and PCR, respectively.

Most of the 45 samples found positive by both PCR and RDT were among male (77.8%), rural (64.4%), and non-Saudi (82.2%) participants; 44.4% were among participants aged 18–30, and 58.5% were in samples with low parasite density. Similar results were found in the 41 samples found positive by both microscopy and PCR. Two RDT-positive samples were considered false positives based on both microscopy and PCR; 16 (4.5%) were considered false negatives among the microscopy-negative samples. Interestingly, 12 PCR-positive samples were found negative by RDT, one of them with moderate parasite density.

**Figure 2:**
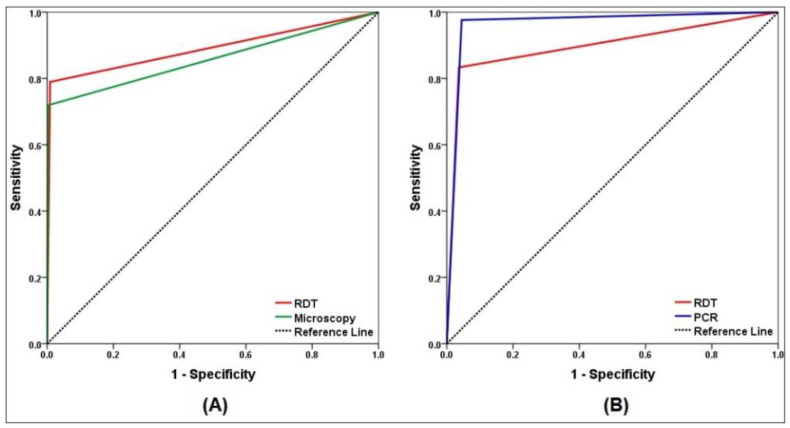
Receiver operating characteristic (ROC) curve analysis of malaria diagnostic techniques. (**A**) ROC for microscopy and RDT versus PCR as reference method. (**B**) ROC for PCR and RDT versus microscopy as reference method.

The comparative performances of the methods are presented in Table 2. Considering PCR as the reference method, the sensitivity and specificity of the RDT were found to be 79% and 99.1%, respectively, while for microscopy, they were 71.9% and 99.7%, respectively. The PLRs of both methods were high, indicating that a positive test can confirm malaria diagnosis. In addition, the NLR of the RDT was 0.20, showing moderate indication that a negative test suggests the absence of malaria. However, the NLR of microscopy was 0.27, suggesting that a negative test does not indicate the absence of malaria. Both the RDT and microscopy strongly agreed with the PCR results (kappa = 0.84 and 0.80, respectively). The AUC of the RDT and microscopy compared with PCR for *P. falciparum* positive cases were 89.0% (95% CI = 82.7–95.4; *p* < 0.001) and 85.8% (95% CI = 78.7–92.9; *p* < 0.001), respectively, indicating outstanding and excellent discriminating ability (Figure 2A).

Considering light microscopy as the reference method, the sensitivity and specificity of the RDT were 83.3% and 96.4%, respectively. However, the sensitivity and specificity of PCR were 97.6% and 95.5%, respectively. The NPVs of the RDT and PCR were high, whereas the PPVs were 72.9% and 71.9%, respectively. The PLRs and NLRs of the RDT were 22.9 and 0.17, and those for PCR were 21.7 and 0.03. The RDT and PCR showed moderate and strong agreement with microscopy (kappa = 0.75 and 0.80, respectively). Compared with microscopy, ROC curves showed outstanding discriminating ability for both RDT and PCR, with AUC of 89.9% (95% CI = 83.1–96.6; *p* < 0.001) and 96.6% (95% CI = 93.6–99.5; *p* < 0.001), respectively (Figure 2B).

The age-stratified performances of the RDT and microscopy for detecting *P. falciparum* were compared with PCR, and the results are shown in Table 3; the RDT showed higher sensitivity than microscopy in all age groups. Interestingly, the performance parameters of the RDT and microscopy decreased as age increased. Both methods were found highly specific in all age groups. The PPVs of the RDT and microscopy were lowest in participants over 40 at 85.7% each. The RDT results strongly agreed with those from PCR in all age groups (kappa values between 0.93 and 0.83), except the over 40 years age group that showed moderate agreement (kappa = 0.67). However, the agreement between microscopy and PCR decreased with age. Strong agreements were found in the below 18 (kappa = 0.93) and 18–30 (kappa = 0.84) age groups, while the agreement was moderate in the older age groups (Table 3).

**Figure 3:**
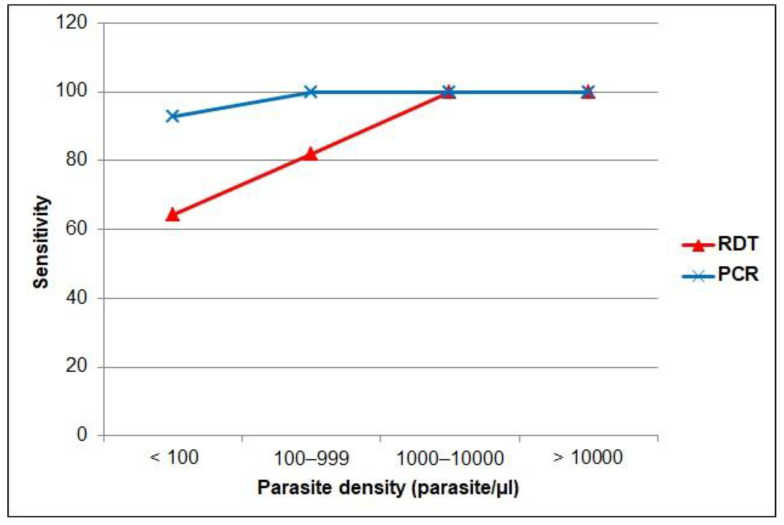
Sensitivity by parasite density level of the RDT and PCR versus microscopy as gold standard.

When the performance was stratified by other characteristics (Table 4), the sensitivity of RDT was comparable in samples collected from men than in those from women. However, the sensitivity of microscopy was slightly higher in rural (80.6%) than urban areas (76.2%). Regarding nationality, the sensitivity of the RDT was higher in samples collected from non-Saudi participants (81.8%) than in those from Saudi (69.2%) participants. Similar results were found for microscopy (75.0% in non-Saudi vs. 61.5% in Saudi). Furthermore, Figure 3 shows that RDT and PCR sensitivity increased consistently with increasing parasite density, with the lowest RDT sensitivity (64.3%; 95% CI = 35.1–87.2) found in samples with a parasite density below 100 parasites/μL. Both RDT and PCR sensitivities were 100% in samples with moderate and severe parasite densities.

## 4. Discussion

An accurate, prompt diagnosis is crucial for managing, controlling, and eliminating malaria. This study evaluated the performance of an RDT, microscopy, and PCR in diagnosing *P. falciparum* malaria among febrile patients in the Jazan region, an area with low transmission. The study identified 60 *P. falciparum* cases, giving a positivity rate of 15.0%. Conventional PCR identified an additional 21.1% (12/57) and 28.1% (16/57) of cases that had been missed by RDT and microscopy, respectively; 8.3% (5/60) were identified only by PCR. Data analysis was performed based on two scenarios considering PCR and microscopy as reference methods.

PCR is highly sensitive when detecting malaria parasites and accurately identifies *Plasmodium* species. It also accurately detects mixed-species infections; thus, it has been widely used for diagnosis, epidemiological surveys, and drug efficacy trials [10,20]. Furthermore, several studies have used nested PCR as a reference method to assess the performance of malaria diagnostics in different settings [20,22,33,34,35,36]. In this study, PCR was superior to microscopy and RDT for diagnosing *P. falciparum* malaria. The results revealed that the diagnostic performance of the AllTest Malaria p.f./p.v. RDT (sensitivity 79%; specificity 99.1%) was better than that of light microscopy (sensitivity 71.9%; specificity 99.7%). The results also showed that the RDT and microscopy had high PPVs and NPVs compared with nested PCR, minimising false positives and negatives. These findings agree with the only previous study in the Jazan region among patients attending King Fahd Central Hospital, which also evaluated the three methods [37]. Hawash et al. [37] reported slightly higher sensitivity (83.3%) and lower specificity (94.2%) for the Paramax-3 RDT (Zephyr Biomedicals, Verna, Goa, India) and slightly higher sensitivity (76.6%) and specificity (100%) for microscopy.

The accuracy of malaria diagnostics depends on several factors, including the level of malaria endemicity, parasite density, mutation or deletion of the gene encoding the HRP2/HRP3, format and type of the RDT product, and storage conditions [17,18,38,39]. Saudi Arabia has not yet eliminated malaria, and Jazan is currently the only region with a limited number of malaria transmission foci and autochthonous cases [25]. Using PCR as a reference method, the sensitivity of the AllTest Malaria p.f./p.v. RDT reported in this study is similar to that reported for the CareStar Malaria p.f/p.v (Pf-HRP2/Pv-pLDH) combo test in low-transmission areas in Ethiopia [17]. However, previous studies in declining malaria transmission or pre-elimination settings demonstrated reduced performance of light microscopy and different Pf-HRP2-based or Pf-HRP2/pLDH combo RDTs [33,40,41,42,43]. On the other hand, a study in a low-transmission region of Senegal reported high sensitivity and specificity for the CareStar Malaria Pf-HRP2/Pv-pLDH combo RDT (97.3% and 94.1%, respectively) and microscopy (93.2% and 100%, respectively) compared with PCR [44]. In contrast, studies in high-transmission settings demonstrated varying performances of microscopy and different commercially available RDTs in detecting *P. falciparum* malaria using PCR as a reference [22,34,35,45,46,47]. Table 5 shows the performance of different RDTs in different malaria transmission settings.

In this study, the AllTest Malaria p.f./p.v. RDT failed to detect 12 (21.1%) positive cases that PCR detected, 7 of which were confirmed by microscopy. This may lead to patients going untreated and becoming parasite carriers and malaria reservoirs in their communities [52]. Such HRP2-based RDT false negative results could be explained by low parasite density (<100 parasite/μL) [19], or HRP2/HRP3 gene deletion or mutation [53]. Therefore, further studies are required to evaluate the HRP2/HRP3 genetic variation and compare the copy number between RDT-positive and RDT-negative samples in the Jazan region. On the other hand, two negative samples were considered RDT false positives, and could have come from patients on antimalarial drugs or who recovered recently. Some studies suggest that HRP2 antigens from an earlier infection persist for several weeks following successful treatment [46]. False positives may lead to unnecessary treatment and divert clinicians’ attention from other fever aetiologies [54]. This issue may reduce the specificity of Pf-HRP2-based RDTs, particularly in high-transmission settings [47,55]; however, this is not the case in low-transmission settings such as Jazan [54]. In addition, RDT false positives due to non-*Plasmodium* infectious agents, such as African Trypanosoma, and immunological factors such as rheumatism have also been reported [55].

**Figure 4:**
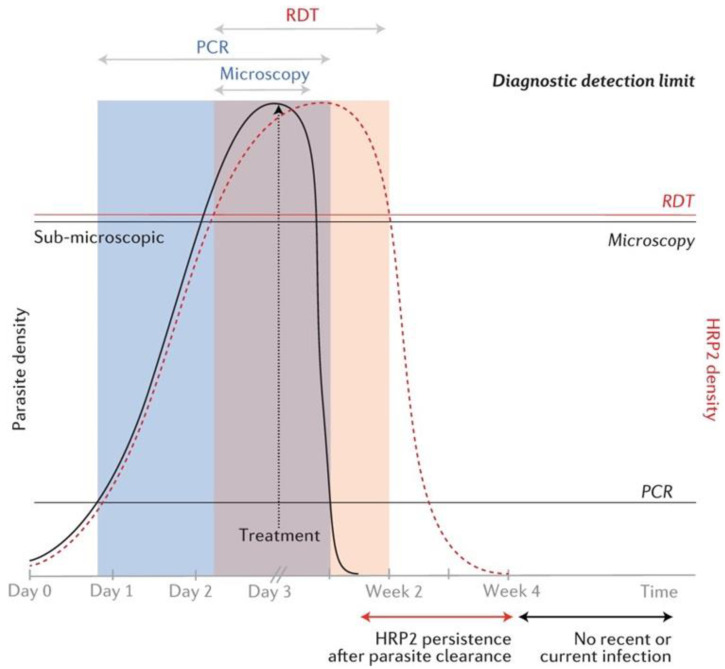
Schematic chart of diagnostic detection limits of microscopy, HRP2-based RDT, and PCR with respect to parasite and HRP2 density. The black line curve represents parasite density and the red dotted curve represents HRP2 gene density. Time scale is in days pre-treatment and in weeks post-treatment. Horizontal lines over the chart represent the detection limits of the three methods respective to parasite and HRP2 densities. The shaded areas represent detectability of parasites by the three methods over time. Reprinted from Wu, L., van den Hoogen, L.L., Slater, H., Walker, P.G., Ghani, A.C., Drakeley, C.J., Okell, L.C. Comparison of diagnostics for the detection of asymptomatic *Plasmodium falciparum* infections to inform control and elimination strategies. *Nature* **2015**, *528* (7580), S86–S93; Copyright (2015). The article is licensed under the Creative Commons Attribution 4.0 International License.

This study showed that microscopy missed 16 (27.6%) positive cases; however, 11 were detected by RDT and treated; microscopy performance is associated with parasite density [18]. A meta-analysis of 42 studies concluded that microscopy missed approximately 50% of PCR-positive malaria cases [56]. Such findings from pre-elimination settings can be attributed to a high proportion of patients with low parasite density and asymptomatic sub-microscopic cases due to immunity acquired when the transmission was higher [45,57]. PCR detectable sub-microscopic infections are sufficient to sustain transmission in the community [58]. Therefore, incorporating more sensitive diagnostic methods may be indispensable in such settings to eliminate residual reservoirs of malaria [59]. A chart of the detection limits of microscopy, HRP2-based RDT, and PCR methods for parasite density and HRP2 genes is shown in Figure 4.

Using microscopy as a reference method, the present study revealed that both RDT and PCR methods performed well in detecting *P. falciparum* parasites. The sensitivity of the AllTest Malaria p.f./p.v. RDT and PCR were 83.3% and 97.6%, respectively, and the specificity was 96.4% and 95.5%, respectively. However, the PPVs of the RDT and PCR were 72.9% and 71.9%, respectively; this indicates that these methods overestimated the positivity rate by approximately 27% and 28%, respectively. Nonetheless, both methods showed PLRs over 10 and NLRs below 0.2, indicating that they are useful diagnostic methods for *P. falciparum* malaria [60]. PPVs and NPVs are affected by the prevalence of malaria in the background, while likelihood ratios are not; therefore, the latter are considered among the best indicators of diagnostic accuracy [60,61]. Although this study suggests that the performance of the AllTest RDT was high, it failed to meet the 95% sensitivity threshold recommended by the WHO [12]. Previous studies in the Jazan region [48] and elsewhere showed varying performance of different commercially available RDTs in detecting *P. falciparum* malaria using microscopy as a reference [33,49,50,51] (Table 5). The performance of RDTs is influenced by product type and format, and the most efficient format depends on the *Plasmodium* species circulating in the targeted area [10]. In general, HRP2-based RDTs demonstrated superior performance when compared with non-HRP2-based RDTs, especially at low parasite densities [12].

This study showed that the performance of RDT and microscopy decreased as age increased, agreeing with previous studies [20,36,47]; this could be explained by age-dependent immunity from frequent exposure [20,62], which could be independent of parasite density [63]. Interestingly, this study showed that RDT and microscopy had significantly higher sensitivity among non-Saudi patients. This finding could be because malaria in Saudi Arabia, including the Jazan region, is mostly imported, with few autochthonous cases reported [25]. In addition, a significant association between parasite density and positive results from three methods was found, and the sensitivity of RDT and PCR increased consistently with increased parasite density. The results also showed that samples with sub-microscopic parasite density increased the probability of false negatives from microscopy. This parasite density-dependent performance was not unexpected; previous studies have shown similar results [12,20,22,31,62,64].

This study had some limitations. First, the data relied on suspected cases in patients presenting at the participating hospitals. This limited the ability to detect asymptomatic, sub-microscopic malaria infections; sub-microscopic infections are predominant in low-transmission settings [57]. Second, the reported positivity rate (14.5%) does not reflect the prevalence of malaria in the Jazan region, which is very low [25]. All positive samples were collected and included in the study, while randomly selected fractions of negative samples from each hospital were included. Third, although the AllTest RDT is designed to detect *P. falciparum* and *P. vivax*, its performance in detecting the latter or mixed infections was not assessed. Fourth, according to the literature, the AllTest RDT has not been assessed by the WHO’s Malaria RDT Product Testing Programme; however, another product (Malaria p.f./Pan Rapid Test Cassette, Product code: IMPN-402) by the same manufacturer was tested and failed to meet the performance criteria [12]. The performance of RDTs is influenced by their brand and format [38]; therefore, these should be considered in any studies on RDT performance, as in this study.

## 5. Conclusions

This study showed that the AllTest Malaria p.f./p.v. Rapid Test Cassette is a useful tool for diagnosing *P. falciparum* malaria in symptomatic patients in the Jazan region, a low-transmission area. Using PCR as a reference method, the AllTest RDT showed better performance than light microscopy and had higher sensitivity and approximately equivalent specificity, PPVs, NPVs, and PLRs, but desirably lower NLRs. However, further studies to evaluate malaria diagnostics among asymptomatic individuals in the region are required.

## Figures and Tables

**Table 1:** Performance matrix of light microscopy and RDT against PCR in the diagnosis of *P. falciparum* malaria according to participants’ characteristics (n = 400).

Characteristic	PCR + ve RDT + ve	PCR + ve RDT − ve	PCR − ve RDT + ve	PCR − ve RDT − ve	PCR + ve LM + ve	PCR + ve LM − ve	PCR − ve LM + ve	PCR − ve LM − ve
Overall	45 (11.2)	12 (3.0)	3 (0.8)	340 (85.0)	41 (10.2)	16 (4.0)	1 (0.3)	342 (85.5)
**Age group (year)**								
<18	8 (17.8)	1 (8.4)	0 (0.0)	39 (11.5)	8 (19.5)	1 (6.2)	0	39 (11.4)
18–30	20 (44.4)	4 (33.3)	1 (33.3)	146 (42.9)	18 (43.9)	6 (37.5)	0	147 (43.0)
31–40	11 (24.4)	3 (25.0)	1 (33.3)	94 (27.7)	9 (22.0)	5 (31.3)	0	95 (27.8)
>40	6 (13.4)	4 (33.3)	1 (33.3)	61 (17.9)	6 (14.6)	4 (25.0)	1 (100)	61 (17.8)
**Sex**								
Women	10 (22.2)	3 (25.0)	0 (0.0)	101 (29.7)	10 (24.4)	3 (18.7)	0	101 (29.5)
Men	35 (77.8)	9 (75.5)	3 (100)	239 (70.3)	31 (75.6)	13 (81.3)	1 (100)	241 (70.5)
**Residence**								
Urban	16 (35.6)	5 (41.7)	1 (33.3)	130 (38.2)	14 (34.1)	7 (43.7)	0	131 (38.3)
Rural	29 (64.4)	7 (58.3)	2 (66.7)	210 (61.8)	27 (65.9)	9 (56.3)	1 (100)	211 (61.7)
**Nationality**								
Saudi	9 (20.0)	4 (33.3)	0 (0.0)	121 (35.6)	8 (19.5)	5 (31.3)	0	121 (35.4)
Non-Saudi	36 (80.0)	8 (66.7)	3 (100)	219 (64.4)	33 (80.5)	11 (68.7)	1 (100)	221 (64.6)

Data are n (%); LM, light microscopy; RDT, rapid diagnostic test.

**Table 2:** Overall performance of light microscopy, RDT, and PCR techniques in the diagnosis of malaria (n = 400).

Test Characteristic	PCR as Reference Method		Microscopy as Reference Method	
	RDT	Microscopy	RDT	PCR
True positive	45	41	35	41
False positive	3	1	13	16
True negative	340	342	345	342
False negative	12	16	7	1
Sensitivity	79.0 (66.1–88.6)	71.9 (58.5–83.0)	83.3 (68.6–93.0)	97.6 (87.4–99.9)
Specificity	99.1 (97.5–99.8)	99.7 (98.4–100)	96.4 (93.9–98.1)	95.5 (92.8–97.4)
PPV	93.8 (82.8–97.9)	97.6 (85.2–99.7)	72.9 (60.8–82.4)	71.9 (61.3–80.6)
NPV	96.6 (94.5–97.9)	95.5 (93.4–97.0)	98.0 (96.2–99.0)	99.7 (98.0–99.9)
PLR	90.1 (29.0–280.7)	239.7 (34.6-1214.3)	22.9 (13.2–49.8)	21.7 (13.8–34.9)
NLR	0.20 (0.13–0.35)	0.27 (0.19–0.43)	0.17 (0.09–0.34)	0.03 (0.0–0.18)
Accuracy	96.3 (93.9–97.9)	95.8 (93.3–97.5)	95.0 (92.4–96.9)	95.8 (93.3–97.5)
*Kappa* value	0.84 (0.76–0.92)	0.80 (0.71–0.90)	0.75 (0.65–0.85)	0.80 (0.71–0.90)

PPV, positive predictive value; NPV, negative predictive value; PLR, positive likelihood ratio; NLR, negative likelihood ratio. Data between parentheses are the 95% CI.

**Table 3:** Age-stratified performance of light microscopy and RDT techniques in the diagnosis of malaria against PCR as reference method.

Method/Age Group	Sensitivity	Specificity	PPV	NPV	*Kappa*
**RDT**					
<18 (n = 48)	88.9 (51.8–99.7)	100.0 (91.0–100)	100.0	97.5 (86.0–99.6)	0.93 (0.77–1.00)
18–30 (n = 171)	83.3 (62.6–95.3)	99.3 (96.3–100)	95.0 (73.8-99.3)	97.3 (93.7–98.9)	0.87 (0.76–0.98)
31–40 (n = 109)	74.6 (49.2–95.3)	98.9 (94.3–99.9)	91.7 (60.6–98.8)	96.9 (92.0–98.8)	0.83 (0.66–0.99)
>40 (n = 72)	60.0 (26.2–87.8)	98.4 (91.3–99.9)	85.7 (44.6–97.8)	93.8 (87.7–97.0)	0.67 (0.40–0.94)
**Microscopy**					
<18 (n = 48)	88.9 (51.8–99.7)	100.0 (91.0–100)	100.0	97.5 (86.0–99.6)	0.93 (0.77–1.00)
18–30 (n = 171)	75.0 (53.3–90.2)	100.0 (97.5–100)	100.0	96.1 (92.5–98.0)	0.84 (0.72–0.97)
31–40 (n = 109)	64.3 (35.1–87.2)	100.0 (96.2–100)	100.0	95.0 (90.4–97.5)	0.76 (0.56–0.96)
>40 (n = 72)	60.0 (26.2–87.8)	98.4 (91.2–99.9)	85.7 (44.6–97.8)	93.9 (87.7–97.0)	0.67 (0.40–0.94)

PPV, positive predictive value; NPV, negative predictive value. Data between parentheses are the 95% CI.

**Table 4:** Performance according to participants’ characteristics of RDT and light microscopy techniques in the diagnosis of falciparum malaria against PCR as reference method.

Characteristic	Method	Sensitivity	Specificity	PPV	NPV	*Kappa*
**Sex**						
Women (n = 114)	RDT	79.9 (46.2–95.0)	100.0 (96.4–100)	100.0	97.1 (92.6–98.9)	0.86 (0.69–1.00)
	Microscopy	76.9 (46.2–94.9)	100.0 (96.4–100)	100.0	97.1 (92.9–98.9)	0.86 (0.69–0.99)
Men (n = 286)	RDT	79.6 (64.7–90.2)	98.8 (96.4–99.7)	92.1 (78.9–97.3)	96.4 (93.7–97.9)	0.83 (0.73–0.92)
	Microscopy	70.5 (54.8–83.2)	99.6 (97.7–100)	96.9 (81.3–99.6)	94.9 (92.1–96.7)	0.79 (0.52–0.95)
**Residence**						
Urban (n = 152)	RDT	76.2 (52.8–91.8)	99.2 (95.8–100)	94.1 (69.1–99.1)	96.3 (92.4–98.2)	0.82 (0.68–0.96)
	Microscopy	66.7 (43.0–85.4)	100.0 (97.2–100)	100.0	94.9 (91.1–97.1)	0.78 (0.62–0.93)
Rural (n = 248)	RDT	80.6 (64.0–91.8)	99.1 (96.6–99.9)	93.6 (78.3–98.3)	96.8 (93.9–98.3)	0.84 (0.74–0.94)
	Microscopy	75.0 (57.8–87.9)	99.5 (97.4–100)	96.4 (79.1–99.5)	95.9 (93.0–97.6)	0.82 (0.72–0.93)
**Nationality**						
Saudi (n = 134)	RDT	69.2 (38.6–90.9)	100.0 (97.0–100)	100.0	96.8 (93.1–98.6)	0.80 (0.62–0.99)
	Microscopy	61.5 (31.6–86.1)	100.0 (97.0–100)	100.0	96.0 (91.5–96.6)	0.74 (0.53–0.96)
Non-Saudi (n = 266)	RDT	81.8 (67.3–91.8)	98.7 (96.1–99.7)	92.3 (79.5–97.4)	96.5 (93.6–98.1)	0.84 (0.75–0.93)
	Microscopy	75.0 (59.7–86.8)	99.6 (97.5–100)	97.1 (82.3–99.6)	95.3 (92.3–97.1)	0.82 (0.72–0.92)

PPV, positive predictive value; NPV, negative predictive value. Data between parentheses are the 95% CI.

**Table 5:** Performance of different RDTs for detection of *P. falciparum* infection at different transmission settings.

RDT Brand	Manufacturer	Target Antigen	Country	Ref. Method	Sen.	Sp.	PPV	NPV	Ref.
**Low-Transmission Settings**									
CareStart	Unlisted	Pf-HRP2/Pan-pLDH	Ethiopia	RT-PCR	67.0	98.5	96.7	86.2	[36]
Paramax-3	Zephyr Biomedicals, India	Pf-HRP2/Pv-pLDH aldolase	Saudi Arabia	Nested PCR	83.3	94.2	64.1	97.8	[37]
Paracheck	Orchid Biomed Systems, India	Pf-HRP2	Zanzibar	RT-PCR	76.5	99.9	96.7	99.0	[41]
First Response	Premier Medical, India	Pf-HRP2/Pan-pLDH	Zanzibar	RT-PCR	64	98.0	72.0	97.0	[42]
CareStar	Access Bio, USA	Pf-HRP2/Pv-pLDH	Senegal	PIET-PCR	97.3	94.1	97.3	94.1	[44]
Binax Now	Alere Scarborough, USA	Pf-HRP2/Pan-aldolase	Saudi Arabia	Microscopy	96.7	78.0	92.5	98.0	[48]
Paracheck	Orchid Biomed Systems, India	Pf-HRP2	Ethiopia	Microscopy	99.4	96.8	91.3	99.8	[49]
CareStar	Access Bio, USA	Pf-HRP2/Pv-pLDH	Ethiopia	Microscopy	99.4	98.0	94.4	99.8	[49]
First Response	Premier Medical, India	Pf-HRP2/Pan-pLDH	Rwanda	Microscopy	80.2	94.3	NA	NA	[33]
**Moderate-to-high transmission settings**									
SD Bioline	Bio Standard Diagnostic, India	Pf-HRP2/Pv-pLDH	India	Nested PCR	82.9	81.5	89.4	71.7	[22]
SD Bioline	Standard Diag., Korea	Pf-HRP2	Sudan	Nested PCR	80.7	89.3	69.2	94.0	[34]
SD Bioline	Standard Diag., Korea	Pf-HRP2/Pan-pLDH	Cameroon	Nested PCR	78	94.0	94.0	78.0	[35]
SD Bioline	Standard Diag., Korea	Pf-HRP2/Pan-pLDH	Tanzania	RT-PCR	75.9	96.9	84.6	94.7	[43]
SD Bioline	Unlisted	Pf-HRP2/Pan-pLDH	Cameroon	Nested PCR	95.3	94.3	97.1	90.9	[46]
SD Bioline	Standard Diag., Korea	Pf-HRP2/Pan-pLDH	Yemen	Nested PCR	96	56.0	76.3	90.4	[47]
ScreenPlus	Unlisted	Pf-HRP2/Pan-pLDH	Indonesia	RT-PCR	75.3	100	100	64.0	[50]
RightSign	Unlisted	Pf-HRP2/Pv-pLDH	Indonesia	Microscopy	100	98.0	98.2	100	[50]
CareStar	Access Bio, USA	Pf-HRP2	Yemen	Microscopy	90.5	96.1	91.0	95.9	[51]

Sen., sensitivity; Sp., specificity; PPV, positive predictive value; NPV, negative predictive value; RT-PCR, real-time PCR; PIET-PCR, photo-induced electron transfer-PCR.

## Data Availability

The data that support the findings of this study are available from the authors upon reasonable request.
